# 49,XXXXY syndrome: A case study and a systematic review of clinical features among the Iranian population

**DOI:** 10.1002/ccr3.6342

**Published:** 2022-09-24

**Authors:** Mahboubeh Rajabzadeh, Nafiseh Taheri, Omid Jazayeri

**Affiliations:** ^1^ Razi Pathobiology Diagnostic Laboratory Department of Genetics Babol Iran; ^2^ Department of Molecular and Cell Biology, Faculty of Science University of Mazandaran Babolsar Iran

**Keywords:** 49,XXXXY syndrome, Fraccaro syndrome, Iranian population, Klinefelter syndrome

## Abstract

The present study describes the clinical, biochemical, hormonal, and developmental characteristics of a patient affected 49,XXXXY syndrome with routine Fraccaro syndrome features accompanied by sexual masturbation behavior. This study summarized the clinical features and also maternal age on birth time of so far 49,XXXXY reported patients among the Iranian population.

## Graphical abstract

There is no association between maternal age and Fraccaro syndrome. Along with classical clinical features of Fraccaro syndrome, our case further presented unusual sexual masturbation behavior. Fraccaro syndrome features in an undiagnosed male should be followed by karyotype analysis and a subsequent appropriate treatment for adequate growth and pubertal development.

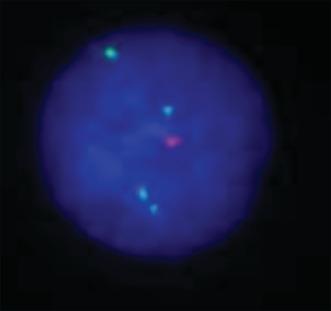

## 1 INTRODUCTION

The human X and Y chromosomes have evolved from a pair of ancestral chromosomes. The X chromosome has retained many properties of an autosome,^1^ containing 2218 genes which is one of the lowest gene density in the human genome (*Homo sapiens* GRCh38.p14). Thus, it is an interesting subject as it has a wide spectrum of clinical manifestations.^2^

49,XXXXY syndrome is a rare sex chromosome polysomy with an approximate incidence of 1 in 85,000 male births.^3^ In 1960, Fraccaro described this clinical entity.^4^ Fraccaro aneuploidy maybe emanates from the nondisjunction of the X chromosome during the meiosis division. These consecutive nondisjunctions will produce an egg with four X chromosomes when fertilized by a Y generation sperm, results in an embryo with 49,XXXXY syndrome.^5^ Interestingly, the appearance of this syndrome does not depend on the age of the mother.^6^ The chromosomal aberration found in Klinefelter syndrome is due to either meiotic or mitotic nondisjunction, leading to a sex‐chromosomal aneuploidy. Two genetic variations exist in Klinefelter syndrome. The majority of cases more than 90% would be presented as a pure form with a 47,XXY karyotype, whereas the remaining 10% include the following sex‐chromosomal abnormalities: Mosaic karyotypes such as 46,XY/47,XXY, higher‐grade aneuploidy such as 48,XXXY; 49,XXXXY, and structurally abnormal X chromosomes.^7^ Klinefelter syndrome might be detected during the prenatal, prepubertal, adolescent, or adult period.^2^ The physical manifestations of Fraccaro syndrome are often variable^8^; Clinical features of Fraccaro syndrome are hyper gonadotropic hypogonadism; moreover, the risk of testicular tumorigenesis and testicular degenerative changes^9^ mental retardation and radioulnar synostosis.^10^ In this study, we report clinical presentation of a 19‐year‐old male patient with Fraccaro syndrome. We also compared all 49,XXXXY syndromes that have been reported in the Iranian population. This study provides new insights into the association between maternal age at birth time and the occurrence of Fraccaro syndrome.

## 2 CASE REPORT

A 19‐year‐old male patient was referred to the Department of Genetics, Razi Pathobiology & Genetic center for karyotyping. He has been the second child of healthy unrelated parents. His mother was 21 years old and his father was 25 years old. There was no evidence of intellectual disability or mental illness in the family. His older sister was a healthy individual with normal development and she has also two normal children.

**FIGURE 1:**
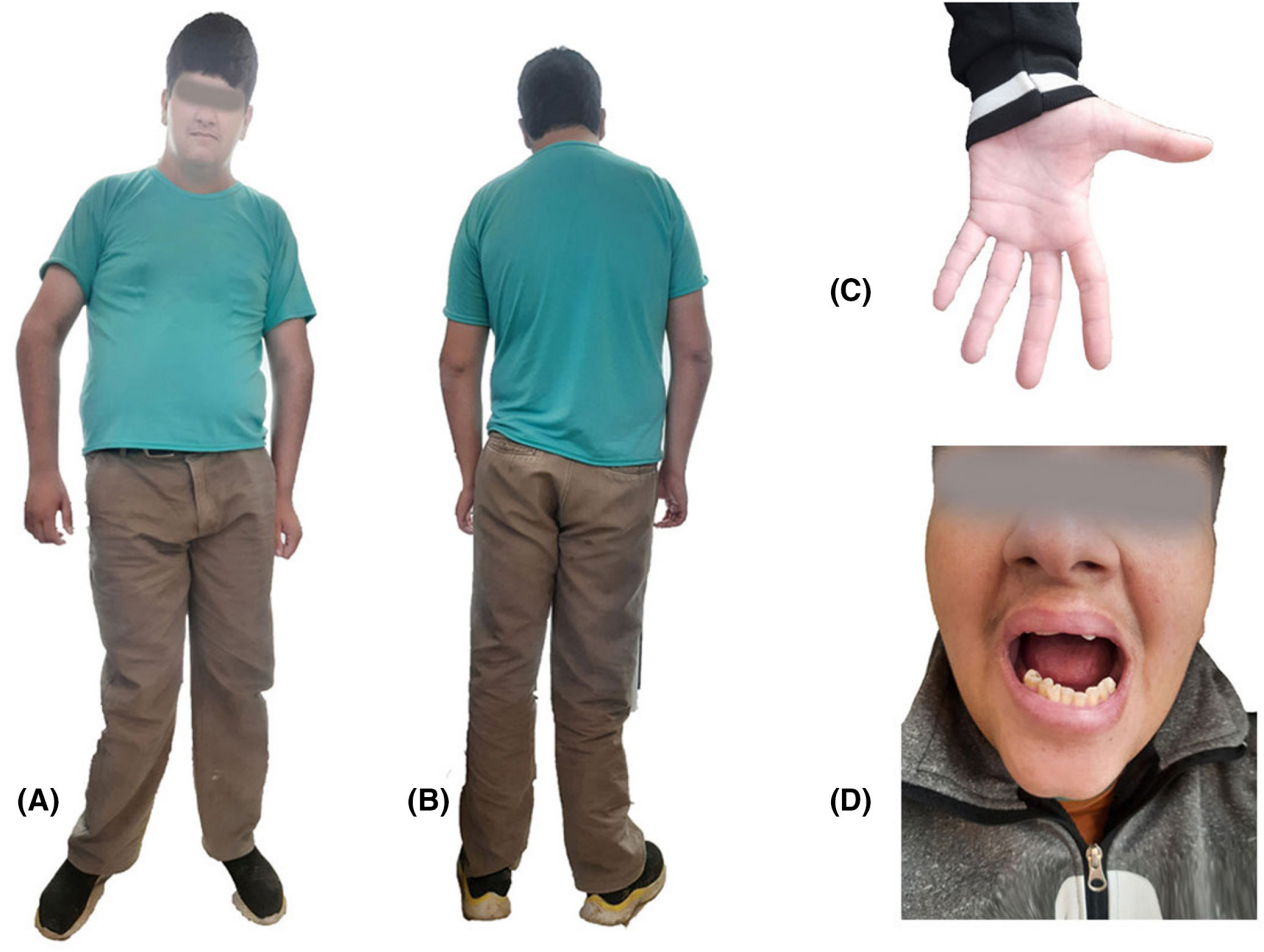
The patient's appearance on frontal (A) dorsal (B), small hands (C) and dental issues (D) views.

The patient's clinical examinations revealed several congenital abnormalities such as specific bone malformation, microcephaly, dental issues, muscular hypotonia, gynecomastia, tall stature disorder, small hands, low nasal bridge, low set ears, unilateral dysplasia of the hip, and azoospermia (Figure 1). The patient had suffered from hypogonadism, micropenis, and masturbation. He also had some learning disabilities, speech impediment which increased the risk of low‐quality friendships and social difficulties in our patient with his peers. In our case, some specific symptoms were detected which includes mild anemia. He also suffered from recurrent bacterial infections during his growth.

## 3 MATERIALS AND METHODS

Peripheral lymphocytes were cultured and GTG‐banded using standard techniques and 50 metaphases were analyzed.

A multiplex quantitative fluorescent polymerase chain reaction (QF‐PCR) was performed using Devyser Compact v3 kit according to the manufacturer's protocol. DNA was extracted from blood samples of the patient using QIAampDSP DNA Blood Mini kit (QIAGEN). The QF‐PCR products were analyzed by capillary electrophoresis on an ABI 3500 automated DNA Sequencer.

Fluorescence in situ hybridization (FISH) analysis of 100 interphase nuclei, using Cytocell probes for chromosomes X, Y and 18 indicated a line proportion for 49,XXXXY (Figure 3). Analysis of the karyotyping and FISH results were performed using GeneASIS 7.2 software (Applied Spectral Imaging) and reported based on the International System for Human Cytogenetic Nomenclature (ISCN)‐2013.

The levels of follicle stimulating hormone (FSH), luteinizing hormone (LH), and total testosterone were measured in the 49,XXXXY patient as he referred to the laboratory, without testosterone therapy. The serum level of LH and FSH were assayed by the LIAISON kits (Diasorin‐Saluggia) and testosterone was also assayed by the Elecsys TESTO II (Roche Diagnostics Gmbl) kits.

To investigate the reported males with Fraccaro syndrome among the Iranian population, we systematically reviewed PubMed, Google scholar, and magiran databases to find related articles in both English and Persian languages, on April 20, 2022. To collect these articles, “Advance search” was used in the PubMed database by applying keywords “Fraccaro Syndrome “and” 49,XXXXY” in (Title/Abstract) combined with “Iran” in (Affiliation). To compile Persian articles, we checked magiran database (http://www.magiran.com) by applying “Fraccaro Syndrome” and “49,XXXXY” as a keyword in (search). Furthermore, Fraccaro Syndrome” and “49,XXXXY” were applied in Google scholar as search terms.

## 4 RESULT

**FIGURE 2:**
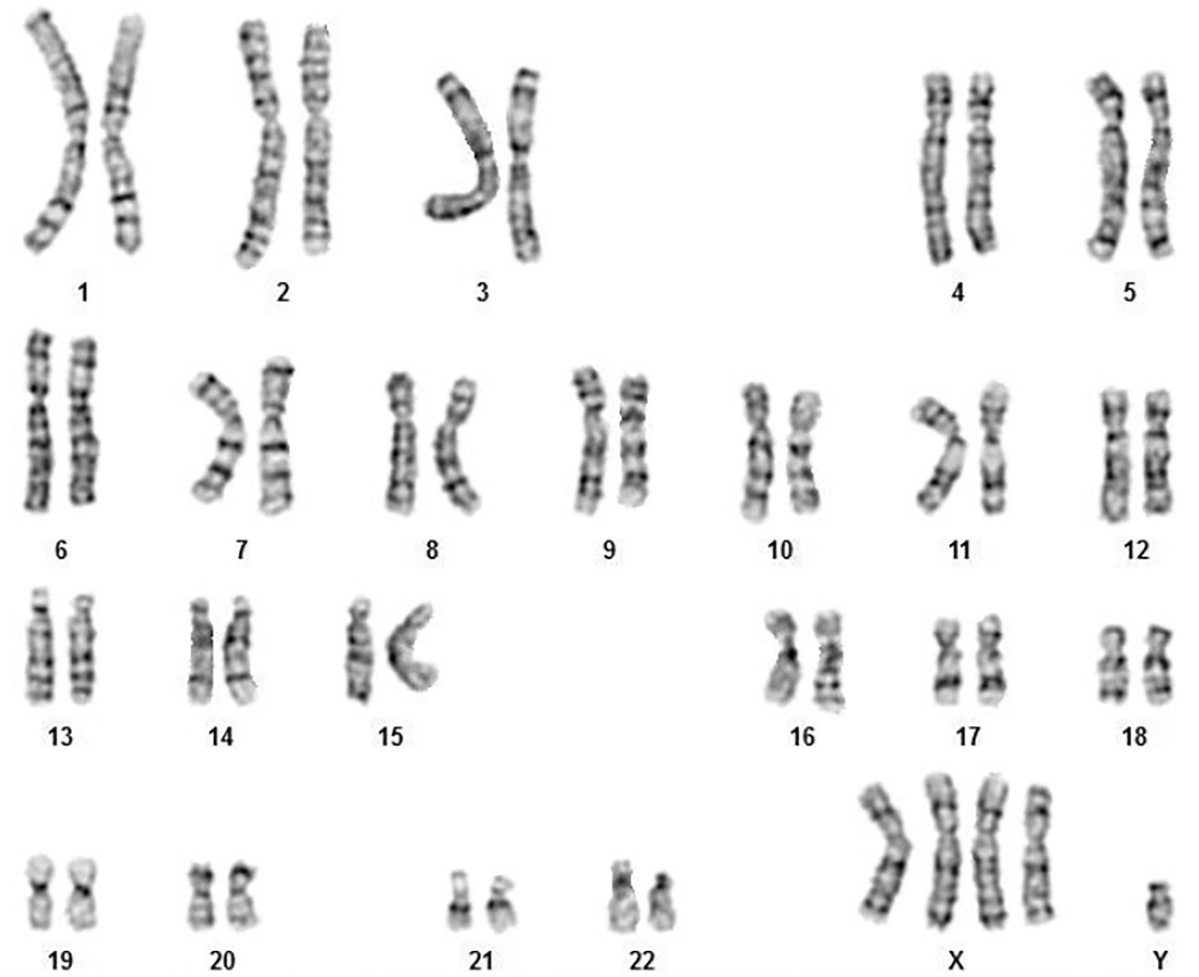
49,XXXXY Karyotype (GTG banding).

**FIGURE 3:**
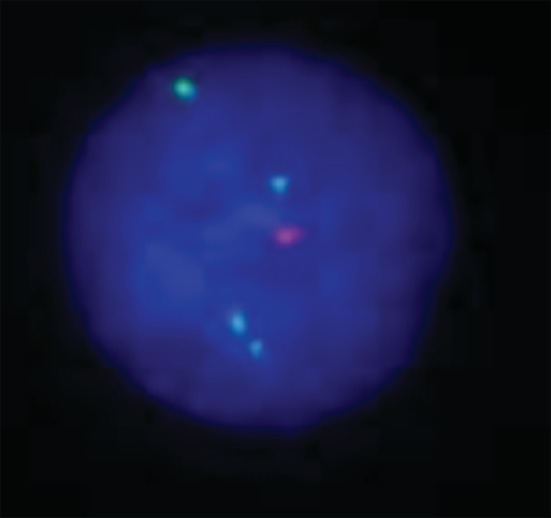
FISH image by using fast prenatal X,Y and 18 Enumeration Probe kit (green signal for X and red signals for Y chromosome).

**FIGURE 4:**
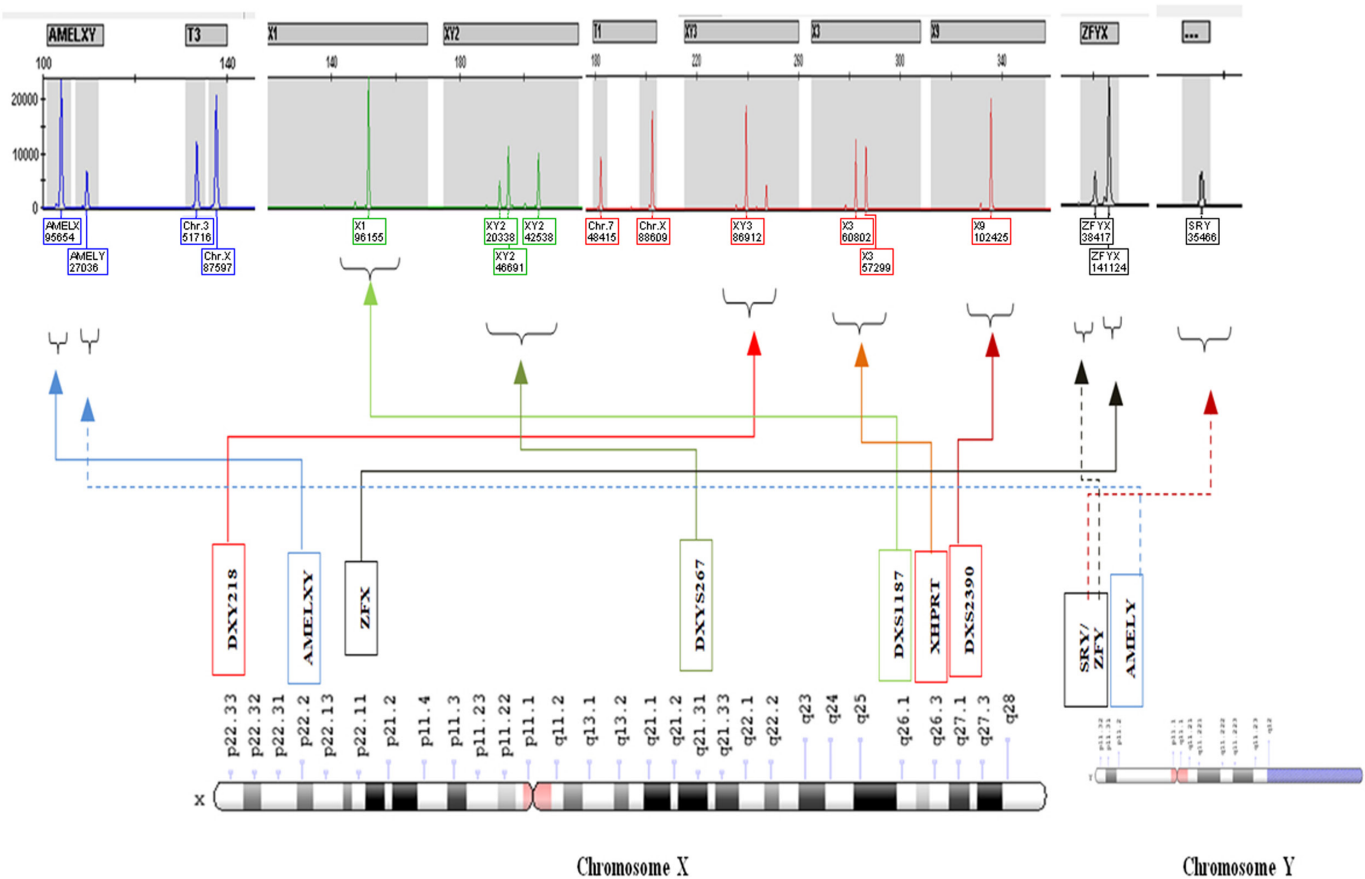
Electrophoretogram showing the QF‐PCR detection of a 49,XXXXY peripheral blood.

In the karyotype study, all metaphases showed a 49,XXXXY syndrome (Figure 2). Investigation of the chromosomal status of the patient's father indicated a normal 46,XY karyotype and it was also the case for his mother 46,XX karyotype. The finding of this karyotype test was also confirmed by both FISH (Figure 3) and QF‐PCR techniques (Figure 4). All short tandem repeat markers for chromosomes 13, 18, and 21 (autosomal) were observed in a normal diallelic pattern. For chromosomes X and Y copy number, the amplification of the homologous gene AMELX/Y indicated a diallelic pattern with an unusual ratio between fluorescent peak areas of 4:1 (Figure 4).

Although the karyotype as a gold standard method, revealed 49,XXXXY in our case, the FISH technique was applied to detect possible low‐level X chromosome mosaicism.^11^ 100 metaphases were studied, and no mosaicism was detected.

The X‐specific peak of the AMELXY is in a ratio of 4:1 if compared with the Y‐specific peak. Electrophoretograms of the QF‐PCR products show four microsatellite markers on chromosomes X (DXS2390, DXS1187, DXYS267, DXYS218, and XHPRT) of the patient. AMELXY and ZFYX (on chromosome X and Y) and SRY (only on chromosome Y) region were used for the detection and comparison of the number of X and Y chromosomes. The box under each peak included molecular size (bp) and the area of the peak. Ratio of the peak areas of AMELXY and DXYS218 had been approximately 4:1 in the patient and indicated the presence of four X and one Y chromosomes.

Results of the serum LH, FSH and testosterone levels had been summarized in Table 1. Additionally, Hemoglobin level was measured 10.6 g/dL; Hematocrit examination revealed dimorphic anemia with lymphocytosis and thrombocytosis. Clinical investigations including serum cholesterol, fasting blood sugar, urine analysis, blood urea nitrogen, ferritin, 25‐OH‐vitamin D, creatinine, calcium, phosphorus, potassium, sodium serum, and alkaline phosphatase levels were measured and all indicated the normal range.

**TABLE 1:** Hormone analysis

Test	Normal range	Test result
LH	2.8–6.8 MIU/ml	14.4 MIU/ml
FSH	1–12 MIU/ml	37.8 MIU/ml
Testosterone	6.68–25.7 nmoL/L	1.51 nmoL/L
GH	Healthy adult male <1.23	0.31 ng/ml

According to the search strategy mentioned in the method section, two articles in Persian (Magiran) and two articles in English (PubMed) were eventually collected and reviewed. Finally, five male patients with Fraccaro syndrome were compiled among the Iranian population (Table 2). This population has been identified in different regions throughout the country that involved cases in Isfahan, Mazandaran, Tehran, and Hamadan provinces.

**TABLE 2:** Reported cases of 49,XXXXY in Iranian population

Reported year	Age of diagnosis	Maternal age	Phenotypic and pathological summary	City	Reference
2010	50 days	24	Hypospadiasis, hypotonia, hypertelorism, atrial septal defect, patient ductus arteriosus, speech impediment	Isfahan	12
2012	11 month	36	Hypogonadism, hypo plastic scrotum, hypothyroidism, hypotonia, low set ears, hypertelorism	Babol	13
2013	45 years	NA	Mild mental retardation and infertility	Tehran	14
2013	10 month	32	Hypotonia, microcephaly, low set ears, micrognathia and congenital heart disease, kyphoscoliosis, clinodactyly of the fourth and fifth fingers of both hands, bilateral clubfoot and unilateral dysplasia of the hip	Tehran	14
2015	2 month	26	Intrauterine growth restriction, low birth weight, facial dysmorphism, Clinodactyly in feet, microphallus and right undescended testis	Hamadan	15
Current study	19 years	21	Hypogonadism, specific bone malformation, microcephaly, dental issues, Muscular hypotonia, behavioral issues, gynecomastia, tall stature, small hands, speech impediment, azoospermia	Babol	‐

Abbreviation: NA, not available.

## 5 DISCUSSION

49,XXXXY is the result of X chromosome mis‐segregation in both meiosis I and meiosis II. Fraccaro's syndrome is caused by 49,XXXXY chromosomal aneuploidy and is often classified as a Klinefelter's syndrome variant. Two prevailing theories have been made to account for the phenotype associated with a 49,XXXXY genotype as well as for the other X chromosome aneuploidies. First, an increased dosages of active genes in regions that escape X inactivation, and second, asynchronous replication of the extra X chromosomes.^16^

The 49,XXXXY syndrome is a very rare but a distinct clinical entity. Typical clinical symptoms include hypogonadism, mental retardation with severe learning difficulties, craniofacial and skeletal abnormalities.^17^, ^18^, ^19^ Along with classical clinical features of Fraccaro syndrome, our case further presented mild anemia, bacterial infections, and sexual masturbation behavior.

The hemoglobin level was in the low range in our patient (10.6 g/dl), but his father had also suffered from a mild anemia. Therefore, anemia is not apparently a clinical symptom of 49,XXXXY syndrome in this case; However, it is worth to mention that a low level of Hemoglobin (10.2 g/dl) had been reported previously in a patient with Fraccaro syndrome.^20^ We could not find any research which addressed the association between hemoglobin level and Fraccaro syndrome. Therefore, this would be an issue for future studies.

In the present study, hormone levels (LH, FSH, and testosterone) were in the abnormal range. This is also in accord with Wei and et al. (2019) findings who obtained the same results.

In Table 2, all would be 49,XXXXY syndrome reported so far in Iran had already been compiled. As far as we know, this study is the first one in which this genetic abnormality type in Iranian population has been compiled. Although the high maternal age would increase the risk of childbirth with chromosomal abnormality and nondisjunction during meiosis,^21^ our finding is somewhat different and is not completely in accordance with the previous results. In fact, maternal age in 3 out of 5 mothers was lower than 30 years. Indeed, our finding is in line with those previous studies which indicated that there is no association between maternal age and Fraccaro syndrome.^22^ Moreover, Lia and colleagues that reported that 49,XXXXY is not associated with maternal age.^23^ These observations differ from Peitsids‘s findings.^24^ Therefore, it is an open question and further studies need to be undertaken in a larger population.

Furthermore, the symptoms including behavioral issues, tall stature with long legs and arms, speech impediments, kidney anomalies, hypogonadism, specific bone malformation and micropenis in an undiagnosed male should be followed by a karyotype analysis and a subsequent appropriate treatment for adequate growth and pubertal development.

## AUTHOR CONTRIBUTIONS

MR performed blood karyotype and participate in manuscript writing. NT performed QF test and analysis. OJ supervised the research, collect the clinical features of patient, and wrote the manuscript.

## CONFLICT OF INTEREST

The authors declare no conflicts of interest.

## CONSENT

Written informed consent was previously obtained from the patient to publish this report in accordance with the journal's patient consent policy.

## Data Availability

Data are available from the corresponding author upon reasonable request.
